# Photoinduced Reversible Deformations of Azobenzene-Based Main-Chain Liquid Crystalline Polymer Films

**DOI:** 10.3390/molecules31203692

**Published:** 2026-10-15

**Authors:** Peng Zhang, Guanfeng Tian, Guojie Wang, Haifeng Yu

**Affiliations:** 1 College of Chemistry and Chemical Engineering, Dezhou University, Dezhou 253023, China; 2 Key Laboratory of Polymer Chemistry and Physics of Ministry of Education, School of Material Science and Engineering, Peking University, Beijing 100871, China; 3 School of Materials Science and Engineering, University of Science and Technology Beijing, Beijing 100083, China

**Keywords:** azobenzene, liquid crystalline polymer, free-standing films, photoactuator

## Abstract

Recently, photodeformable liquid crystalline polymer materials (LCPMs) have attracted tremendous attention owing to their fascinating photoresponsive properties and promising applications. However, conventional photodeformable LCPMs invariably depend on mesogenic alignment and irreversible crosslinking, which conflate structural order with chemical irreversibility and leave no window for post-curing manipulation. To overcome this fundamental limitation, an azobenzene-containing main-chain LCP was synthesized via stepwise polymerization, and free-standing films were subsequently prepared by a simple drop-coating method, without any pre-alignment or crosslinking steps during film formation. Upon 365 nm ultraviolet (UV) irradiation (110 mW cm^−2^), azobenzenes in the LCP undergo trans-to-cis isomerization, resulting in a significant decrease in the storage modulus (*G*′). Subsequent 530 nm green light exposure (110 mW cm^−2^) induces the reverse cis-to-trans isomerization, leading to an increase in *G*′. Furthermore, LCP films exhibit rapid and reversible deformations upon alternating exposure to 365 nm (110 mW cm^−2^) and 530 nm (110 mW cm^−2^) light, with a bending-flattening cycle completed within 50 s. This strategy offers a simple and general route to polydomain, non-oriented free-standing LCP films capable of reversible photoactuation, showing promise for soft actuators and light-driven devices.

## 1. Introduction

As a prominent class of smart materials, shape-changing materials have long been a research focus in the field of stimuli-responsive systems [1,2,3,4]. Among these, the materials capable of reversible deformations are particularly attractive due to their excellent reusability and broad application potentials, with LCPMs being a prime example [5,6]. LCPMs—primarily LC elastomers (LCEs) and LC networks (LCNs)—undergo macroscopic deformations upon the loss of mesogenic order and recover their original shapes when mesogen alignment is restored [7]. Remarkably, such reversible deformations can be cycled almost indefinitely [8]. Currently, the majority of LCPMs are responsive to thermal [9,10], electrical [11], magnetic [12,13], or light stimuli [14,15,16,17]. Unlike the traditional stimuli (heat, electricity, magnetism) that generally require direct contact or elaborate external devices, light enables remote, non-contact actuation with high spatiotemporal precision and facile wavelength tunability [18,19]. Moreover, photo-driven LCPMs can directly convert light energy into mechanical work upon light irradiation, rendering them particularly promising in soft functional devices [20,21,22].

To date, two main strategies have been adopted to endow LCPMs with reversible photoactuation. The first approach involves incorporating photothermal reagents into single-domain LCEs [23]. Upon light exposure, these reagents absorb photons and convert them into heat, raising the local temperature above the LC-to-isotropic phase transition temperature (*T_i_*) and triggering a transition to the isotropic state, which induces macroscopic deformations of the LCEs. Upon removal of light, these LCEs passively cool and fully return to their initial states [24,25]. The second strategy relies on grafting photochromic moieties into single-domain LCEs or LCNs. Azobenzene, a prototypical light-responsive chromophore, has been extensively employed in LCPMs [26,27,28]. Upon alternating irradiation with UV and visible light, azobenzene undergoes reversible trans-cis isomerization accompanied by changes in molecular morphologies [29,30,31]. When azobenzene chromophores are incorporated into single-domain LCEs or LCNs, these reversible molecular length changes are amplified into macroscopic deformations [32,33,34]. Despite remarkable advances in photodeformable LCPMs, the field remains constrained by a deeply entrenched paradigm—that high-performance actuation necessitates both mesogen alignment and covalent crosslinking—a dual requirement that imposes substantial processing complexity and inherently sacrifices reprocessability.

To address the issue, we synthesized an azobenzene-based main-chain LCP by stepwise polymerization, and free-standing films were prepared by casting the LCP solution onto Teflon substrates, without any pre-alignment or crosslinking steps during film formation. Upon 365 nm UV irradiation (110 mW cm^−2^), the LCP films bent towards the light source. Subsequently, upon exposure to 530 nm green light (110 mW cm^−2^), these bent films reverted to their original flat shapes. Upon alternating irradiation with 365 nm UV (110 mW cm^−2^) and 530 nm green light (110 mW cm^−2^), the films displayed reversible bending and flattening. Furthermore, we systematically investigated the influence of light intensity and film thickness on the light-induced deformations of the LCP films. The excellent photoactuation performance endows these films with potential applications in miniaturized functional devices.

## 2. Results and Discussion

### 2.1. Synthesis and Characterization of the Azobenzene-Based LCP

**Scheme 1:**
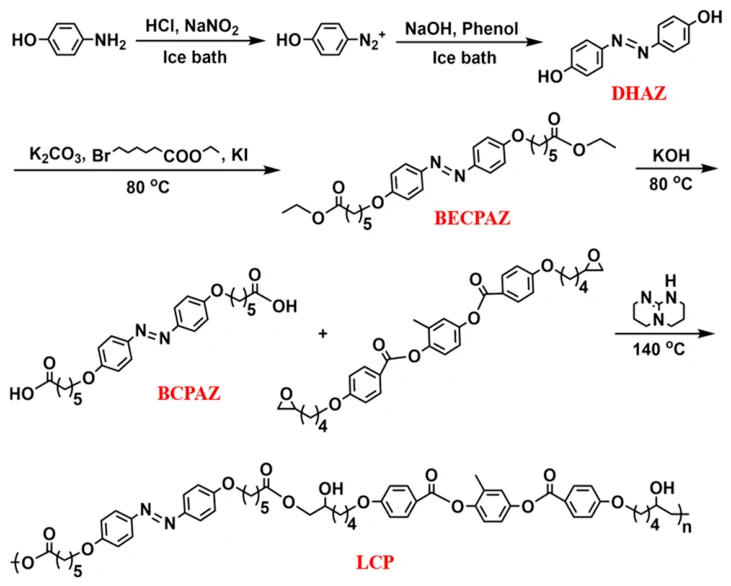
Synthetic route to the azobenzene-based LCP.

The azobenzene-containing LCP used in this work was synthesized as reported in Ref. [35] (same batch); its synthetic route is shown in Scheme 1. First, 4,4′-dihydroxyazobenzene (DHAZ) was synthesized from *p*-aminophenol via a diazo coupling reaction. Subsequently, DHAZ underwent nucleophilic substitution with ethyl 6-bromohexanoate to afford 4,4′-bis[5-(ethoxycarbonyl)pentyloxy]azobenzene (BECPAZ), which was hydrolyzed under alkaline conditions to yield 4,4′-bis[5-(carboxy)pentyloxy]azobenzene (BCPAZ). Finally, the LCP was synthesized by stepwise polymerization of BCPAZ with 2-methyl-1,4-bis[4-(oxirane butoxy)benzoyloxy]benzene, using 1,5,7-triazabicyclo[4,4,0]dec-5-ene as a catalyst.

**Figure 1:**
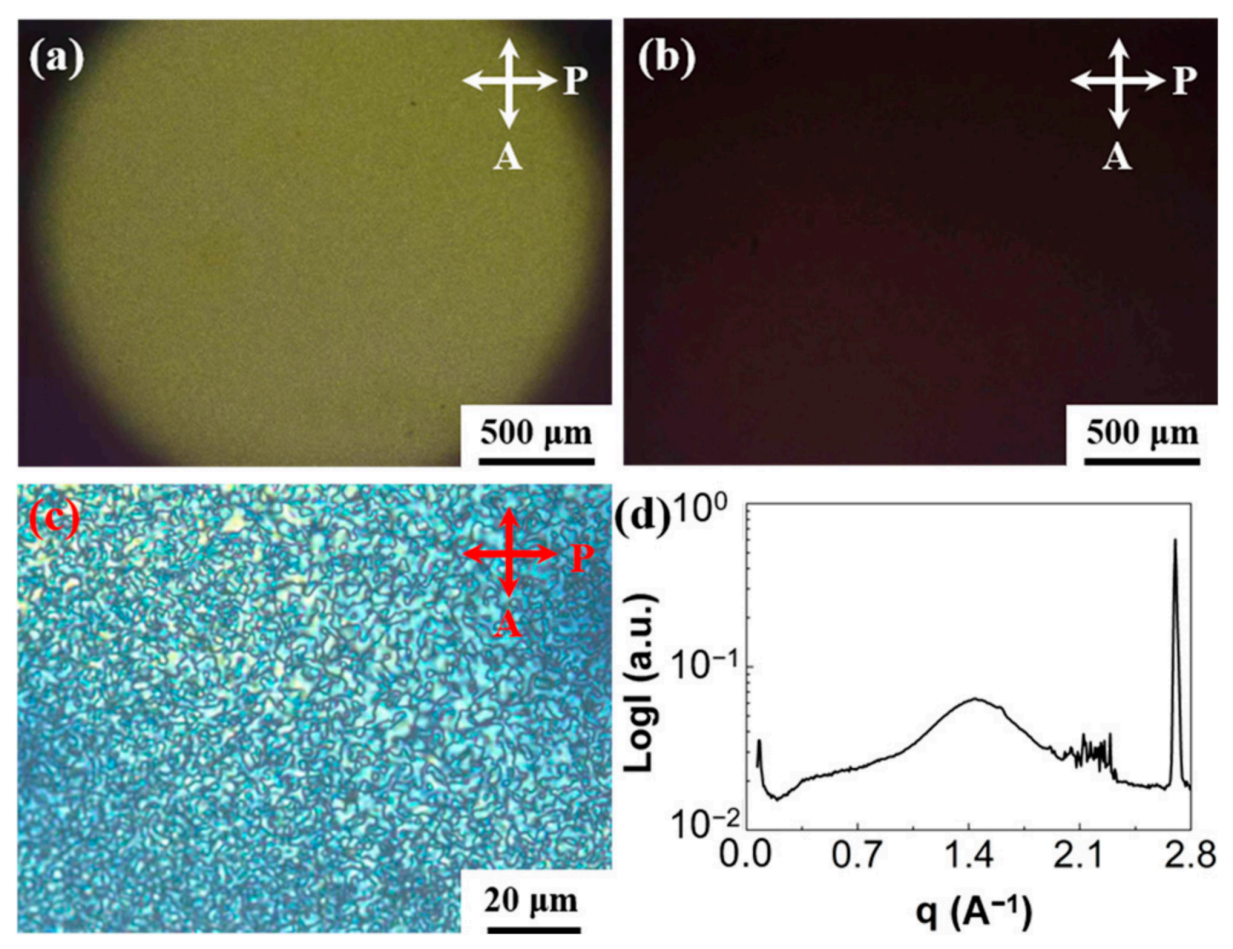
POM images of the azobenzene-based LCP at (**a**) 25 °C, (**b**) 140 °C, and (**c**) 65 °C. Solid double arrows indicate the polarization directions of the polarizer (P) and analyzer (A). (**d**) WAXS pattern of the LCP.

The azobenzene-containing LCP has a number-average molecular weight (*M_n_*) of 2.5 × 10^4^ g mol^−1^ and a polydispersity index (PDI) of 3.3 [35], corresponding to a degree of polymerization of approximately 26. The glass-transition temperature (*T_g_*) and *T_i_* of the LCP are 45.8 °C and 85.0 °C, respectively [35]. To further study its LC behavior, 5 mg of the LCP was dissolved in 0.3 mL of N,N-dimethylformamide (DMF) and drop-cast onto a glass slide. The sample was first kept at room temperature for 10 h to allow preliminary solvent evaporation and then dried at 80 °C for an additional 10 h. After cooling to room temperature, the sample was placed under a polarizing optical microscope (POM) equipped with a heating stage. At 25 °C, birefringent domains were observed between two crossed polarizers (Figure 1a). Upon heating to 140 °C at 10 °C min^−1^, the LCP became isotropic and exhibited a dark image under crossed polarizers (Figure 1b). After annealing at 140 °C for 20 min, the sample was slowly cooled to 65 °C at 0.5 °C min^−1^, and a schlieren texture characteristic of nematic LCs was observed (Figure 1c). The LC structure of the LCP was further confirmed by X-ray scattering. No diffraction peaks appeared in the small- and medium-angle regions, while a broad diffuse peak was observed in the wide-angle region (Figure 1d), consistent with a nematic LC phase. Based on the above results, the azobenzene-containing LCP is assigned as a nematic LC polymer.

### 2.2. Photoresponsive Behaviors of the Azobenzene-Based LCP

**Figure 2:**
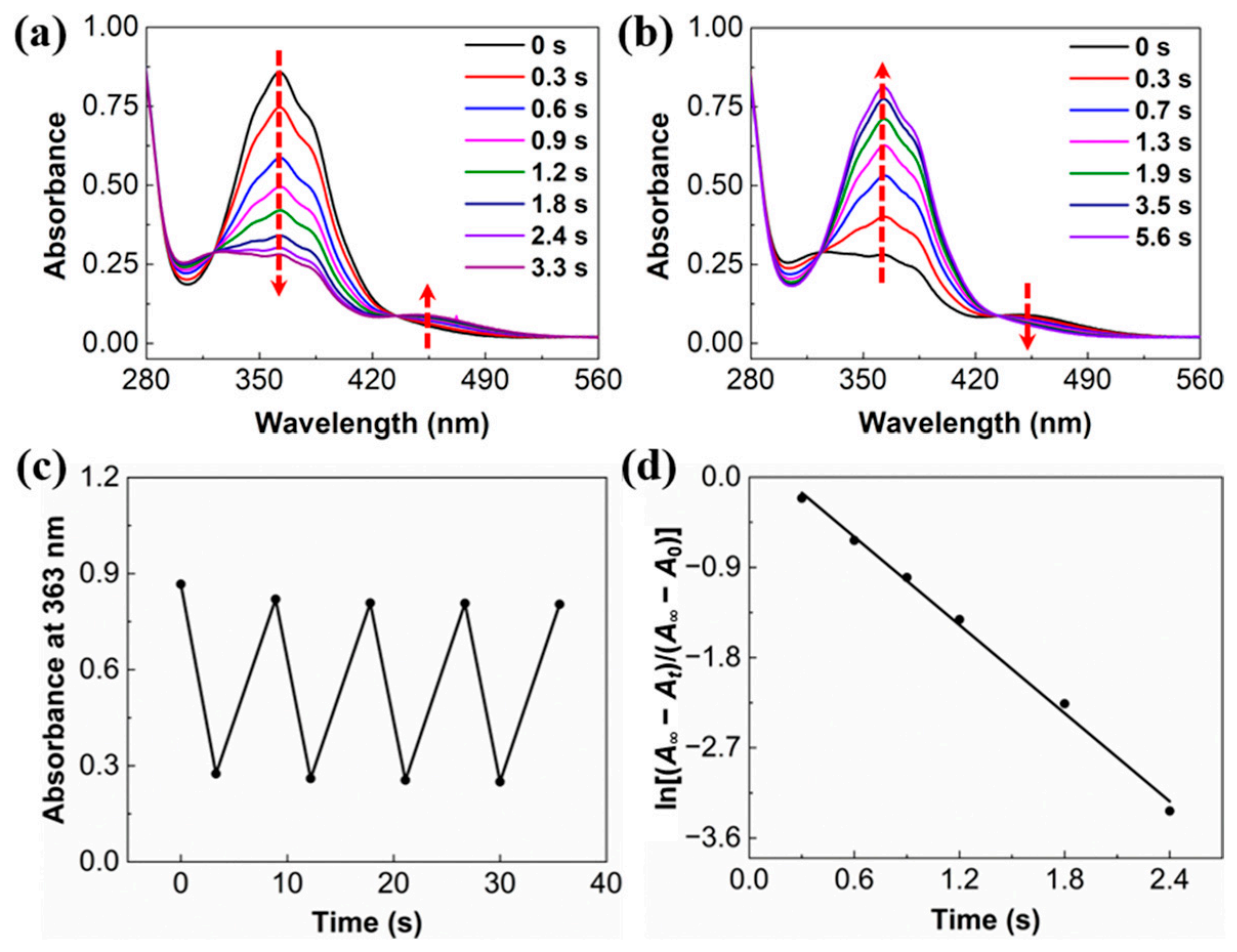
UV-visible absorption spectra of the azobenzene-based LCP upon sequential exposure to (**a**) 365 nm UV (110 mW cm^−2^) and (**b**) 530 nm green light (110 mW cm^−2^). (**c**) Changes in the absorbance at 363 nm of the LCP upon alternating irradiation with 365 nm UV (110 mW cm^−2^) and 530 nm green light (110 mW cm^−2^). (**d**) First-order kinetic plot of ln[(*A*_∞_ − *A_t_*)/(*A*_∞_ − *A*_0_)] versus time.

A 4 wt% solution of the LCP in tetrahydrofuran (THF) was spin-coated onto quartz substrates at 3000 rpm for 20 s and then dried at room temperature for 10 h. Subsequently, the photoisomerization behavior of the LCP was investigated using a UV/VIS spectrophotometer. Before irradiation, the LCP displayed a strong π-π* absorption band centered at 363 nm and a weak n-π* band at 448 nm, indicating that the azobenzene moieties were predominantly in the trans state. Upon irradiation with 365 nm UV light (110 mW cm^−2^), the absorbance at 363 nm gradually decreased while that at 448 nm increased (Figure 2a), evidencing the trans-to-cis isomerization of the LCP. When the film was thereafter irradiated with 530 nm green light (110 mW cm^−2^), the absorbance at 363 nm gradually increased while that at 448 nm decreased (Figure 2b), indicating the cis-to-trans back-isomerization of the LCP. A photostationary state (PSS) was obtained at 5.6 s with ~5.4% cis content remaining [36]. Notably, the 363 nm absorbance at this PSS was lower than that of the pristine film, indicating incomplete back-conversion. Upon alternating irradiation with 365 nm UV (110 mW cm^−2^) and 530 nm green light (110 mW cm^−2^), the LCP underwent reversible trans-cis isomerization (Figure 2c). By contrast, direct irradiation of the pristine LCP film with 530 nm green light (110 mW·cm^−2^) caused no change in the 363 or 448 nm absorbances (Figure S1), confirming that 530 nm green light alone does not induce the trans-to-cis isomerization of the LCP. Upon 365 nm UV irradiation (110 mW cm^−2^), the trans-to-cis isomerization process of the LCP followed an apparent first-order law:

ln[(*A*_∞_ − *A_t_*)/(*A*_∞_ − *A*_0_)] = −*kt* (1)

where *A*_0_, *A_t_*, and *A*_∞_ are the absorbances at 363 nm before irradiation, at time *t*, and at the photostationary state, respectively, and *k* is the rate constant. The linear fit of Equation (1) is shown in Figure 2d. From this fit, *k* = 1.45 ± 0.04 s^−1^ (R^2^ = 0.996).

To further investigate the photoresponsive behaviors of the azobenzene-based LCP, differential scanning calorimetry (DSC) was employed to evaluate its thermal transitions. Prior to irradiation, the LCP exhibited a *T_g_* of 45.8 °C [35], well above room temperature. After irradiation with 365 nm UV light (110 mW cm^−2^), the *T_g_* dropped to −15.8 ± 4.6 °C (mean ± standard deviation (SD), *n* = 3; Figure S2), falling well below ambient. This significant decrease in *T_g_* can be attributed to the weakening of intermolecular interactions upon trans-to-cis isomerization of the LCP, consistent with Jenekhe et al., who demonstrated that a reduction in intermolecular forces markedly lowers the *T_g_* of polymer systems [37]. In addition, a photoinduced LC-to-isotropic phase transition also occurred because the cis-azobenzene often destabilized the mesogenic phase, which further contributes to the decrease in *T_g_* of the polymer [38]. Compared with the rod-like trans isomer, the bent cis geometry of azobenzene disrupts regular chain packing and weakens intermolecular cohesive interactions, which enhances chain mobility and thereby substantially lowers *T_g_* [39].

**Figure 3:**
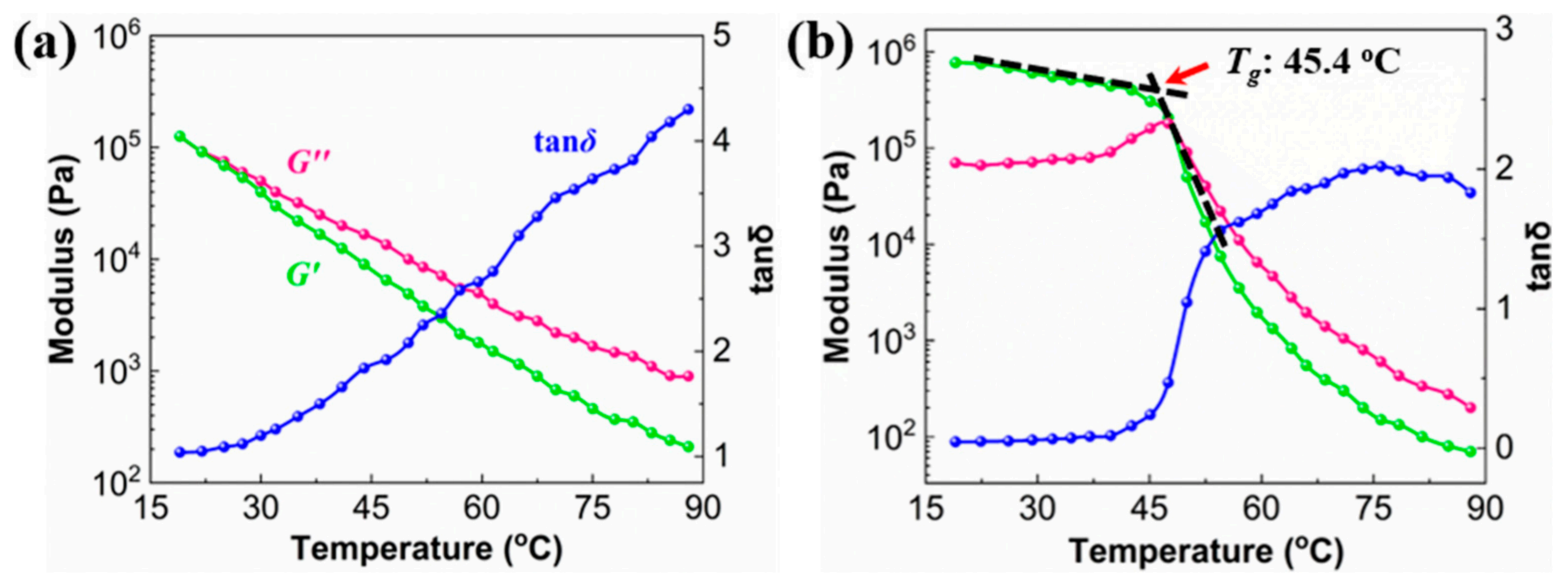
Temperature-dependent *G*′, *G*″, and tan *δ* of the azobenzene-based LCP after sequential irradiation with (**a**) 365 nm UV (110 mW cm^−2^) and (**b**) 530 nm green light (110 mW cm^−2^).

Dynamic mechanical analysis (DMA) of the LCP was performed using a rotational rheometer. Before irradiation, *G*′ was 1.7 × 10^7^ Pa at 25 °C [35]. No distinct peak was observed in the loss factor (tan *δ*); therefore, the *T_g_* of the LCP was determined from the onset point of the *G*′ drop (intersection of tangents), giving 46.8 °C, which is in good agreement with the DSC result (45.8 °C) [35]. After irradiation with 365 nm UV light (110 mW cm^−2^), *G*′ dropped sharply from 1.7 × 10^7^ to 6.9 × 10^4^ Pa at 25 °C, with loss modulus *G*″ > *G*′ and tan *δ* > 1 (Figure 3a), indicating a transition to viscous-dominant behavior. Thus, UV-induced trans-to-cis isomerization substantially softened the polymer. Subsequent exposure to 530 nm green light (110 mW cm^−2^) increased *G*′ to 7.2 × 10^5^ Pa at 25 °C, where *G*′ > *G*″ and tan *δ* < 0.1 (Figure 3b), confirming that cis-to-trans back-isomerization partially restored the elastic modulus. Notably, after 530 nm green light exposure, spectroscopic recovery was nearly complete (Figure 2b) because cis-to-trans back-isomerization largely restored the azobenzene chromophore’s absorption, even though ~5.4% cis remains. In contrast, mechanical recovery is far less complete. To further investigate this discrepancy, post-green-light *T_g_* was measured by DSC. The DSC curve shows two distinct *T_g_* values: 0.9 °C and 48.5 °C (Figure S3). The higher *T_g_* corresponds to the trans-state polymer, whereas the lower *T_g_* is assigned to a mixed cis/trans phase that did not fully relax to the pristine trans conformation. No clear *T_i_* was observed, indicating partial disruption of mesogen packing. The DSC data reveal that spectroscopic and mechanical recovery probe different length scales. UV-vis probes the azobenzene chromophore configuration, which recovers ~95%. However, the macroscopic modulus depends not only on the isomer ratio but also on chain packing, π-π stacking, and other supramolecular aggregate structures, which do not immediately return even when individual chromophores have isomerized back to trans. The biphasic *T_g_* confirms that a fraction of the azobenzene-based polymer retains a cis/trans mixed morphology with a much lower *T_g_* (~0.9 °C), acting as a soft phase that depresses the overall modulus.

### 2.3. Photoinduced Reversible Deformations of Azobenzene-Based LCP Films

**Figure 4:**
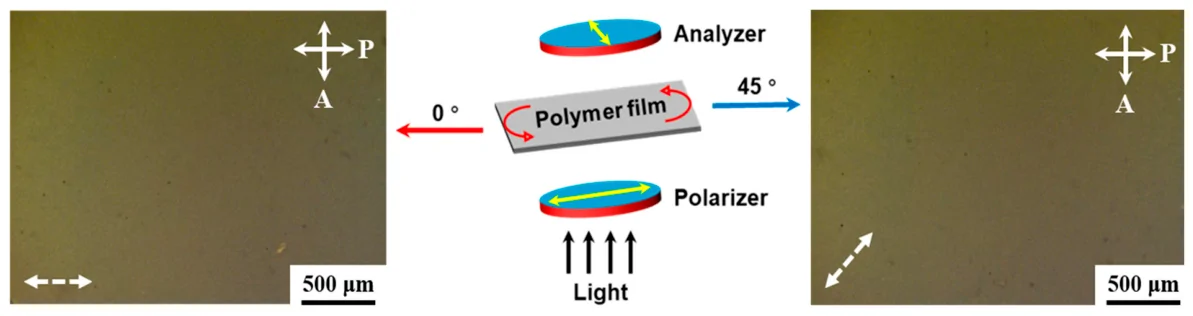
POM images of the azobenzene-based LCP film. Solid double arrows indicate the polarization directions of the polarizer (P) and analyzer (A), and dashed double arrows represent the long-axis directions of the film.

Free-standing azobenzene-containing LCP films were prepared by a simple drop-coating method, without any pre-alignment or crosslinking steps during film formation. As shown in Scheme S1, the LCP was dissolved in DMF and uniformly drop-cast onto Teflon substrates. After solvent evaporation, brownish-yellow films (~15 μm thickness) were obtained by mechanical peeling from the substrates. To examine whether the azobenzene moieties were aligned during film formation, the film sample was observed under a POM between two crossed polarizers. Upon rotation, no alternating bright-dark contrast was observed (Figure 4), confirming that the azobenzene mesogens remained randomly distributed in the films.

**Figure 5:**
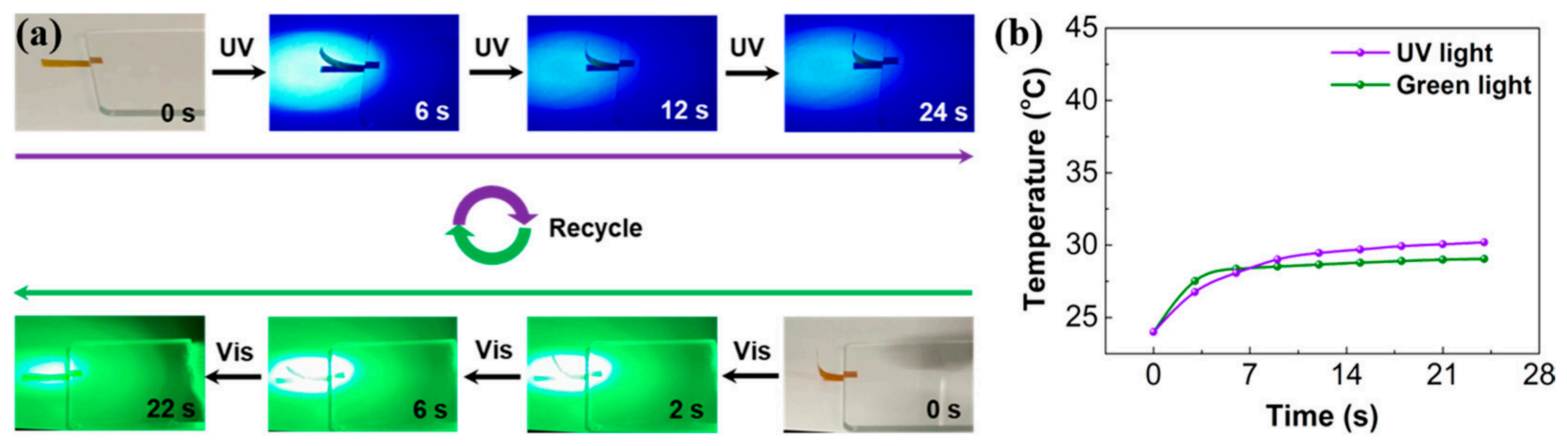
(**a**) Upon sequential irradiation with 365 nm UV (110 mW cm^−2^) and 530 nm green light (110 mW cm^−2^), the azobenzene-based LCP film bends towards the incident light and returns to its original flat shape. Film dimensions: 10 mm × 2 mm × 15 μm (length × width × thickness). (**b**) Surface temperatures of the LCP film as a function of time upon sequential exposure to 365 nm UV (110 mW cm^−2^) and 530 nm green light (110 mW cm^−2^).

As shown in Figure 5a, upon 365 nm UV irradiation (110 mW cm^−2^) of the top surface, the LCP film bent towards the incident light and reached a maximum deflection within 24 s, after which the deflection remained nearly constant upon further irradiation (Video S1). Subsequent 530 nm green light exposure (110 mW cm^−2^) gradually reduced the deflection, and the bent film nearly returned to its initial flat shape within 22 s (Video S2). When the bottom surface was sequentially irradiated with 365 nm UV (110 mW cm^−2^) and 530 nm green light (110 mW cm^−2^), the film bent towards the light source and then nearly reverted to its original flat state (Figure S4). Under the same conditions, the surface temperatures of the film rose from room temperature to only 30.2 °C (UV) and 29.1 °C (green light, Figure 5b), both far below the *T_g_* of the LCP (45.8 °C). In addition, when pristine LCP films were heated to 35 °C in the dark with either the top or bottom surface in contact with a heat source, no bending was observed (Figure S5), confirming that the photothermal contribution is negligible. Furthermore, since the azobenzene mesogens in the LCP films are non-oriented (Figure 4), the bending deformation of the film cannot originate from the orientation-driven contraction. Moreover, although the trans-to-cis isomerization of azobenzene is known to increase free volume and generally drives the films to bend away from the light source [40], the present LCP films are bent towards the incident light, eliminating the free-volume expansion mechanism. Recently, Wu and co-workers reported UV-induced bending in an azobenzene-containing polymer film, proposing that a gradient in *T_g_* across the film thickness—caused by uneven cis-isomer accumulation—governs the photomechanical response [41]. For the azobenzene-containing LCP films, upon 365 nm UV exposure, the irradiated surface of the film absorbs photons first. Due to the large molar extinction coefficient of the azobenzene moieties, photon flux decays through the film thickness, inducing the trans-to-cis isomerization of the azobenzene moieties predominantly at the illuminated surface: (i) reducing the molecular aspect ratio of azobenzene at the illuminated surface, which generates local in-plane contraction strain and tends to contract the surface layer; (ii) decreasing the *T_g_* and elastic modulus locally at the irradiated top surface relative to the bottom, generating an asymmetric stress profile (softer top/stiffer bottom) that drives the film to bend towards the light source. Subsequent 530 nm green light irradiation induces the cis-to-trans back-isomerization of the azobenzene moieties at the irradiated surface, raising the local modulus and allowing the film to flatten. To sum up, the bending deformations of the azobenzene-containing LCP films arise from two coupled mechanisms: photoinduced contraction strain at the irradiated surface and a through-thickness *T_g_* and modulus gradient.

**Figure 6:**
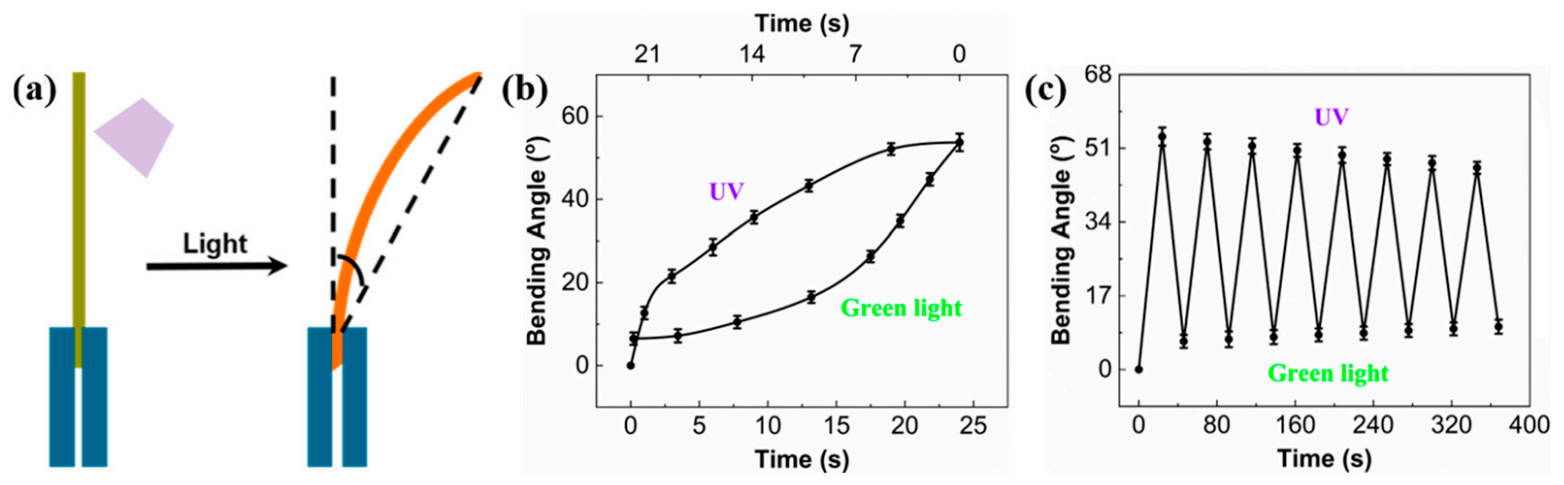
(**a**) Schematic illustration of the bending angle of the azobenzene-based LCP film. (**b**) Upon sequential exposure to 365 nm UV (110 mW cm^−2^) and 530 nm green light (110 mW cm^−2^), the LCP film reaches a maximum bending angle of 53.7 ± 2.1° and recovers to a residual angle of 6.5 ± 1.5°. (**c**) Changes in the bending angle of the LCP film upon alternating irradiation with 365 nm UV (110 mW cm^−2^) and 530 nm green light (110 mW cm^−2^).

Bending angle was used to quantitatively evaluate the degree of photoinduced deformation, defined as the angle swept by the film tip (Figure 6a). Upon 365 nm UV irradiation (110 mW cm^−2^), the maximum bending angle of the azobenzene-based LCP film reached 53.7 ± 2.1° (mean ± SD, *n* = 3) (Figure 6b). Subsequent exposure to 530 nm green light (110 mW cm^−2^) decreased the bending angle to 6.5 ± 1.5° (mean ± SD, *n* = 3), indicating nearly complete recovery to the initial flat state. Furthermore, upon alternating exposure to 365 nm UV (110 mW cm^−2^) and 530 nm green light (110 mW cm^−2^), the LCP film underwent reversible bending-flattening cycles over multiple iterations (Figure 6c). With increasing cycle number, the maximum bending angle decreased from ~53.7° to ~46.5°, while the residual angle increased from ~6.5° to ~9.9°. Consequently, the actuation amplitude—defined as the difference between the maximum and residual bending angles—gradually decreased, with ~78% retention of the initial value after 8 cycles. The large bending angle and repeated stress caused progressive film fatigue, and beyond 8 cycles the residual angle became too large to allow meaningful comparison. This behavior is consistent with the incomplete cis-to-trans recovery observed in the UV-vis absorption spectrum (Figure 2b).

### 2.4. Load-Bearing Capacity of LCP Films

**Figure 7:**
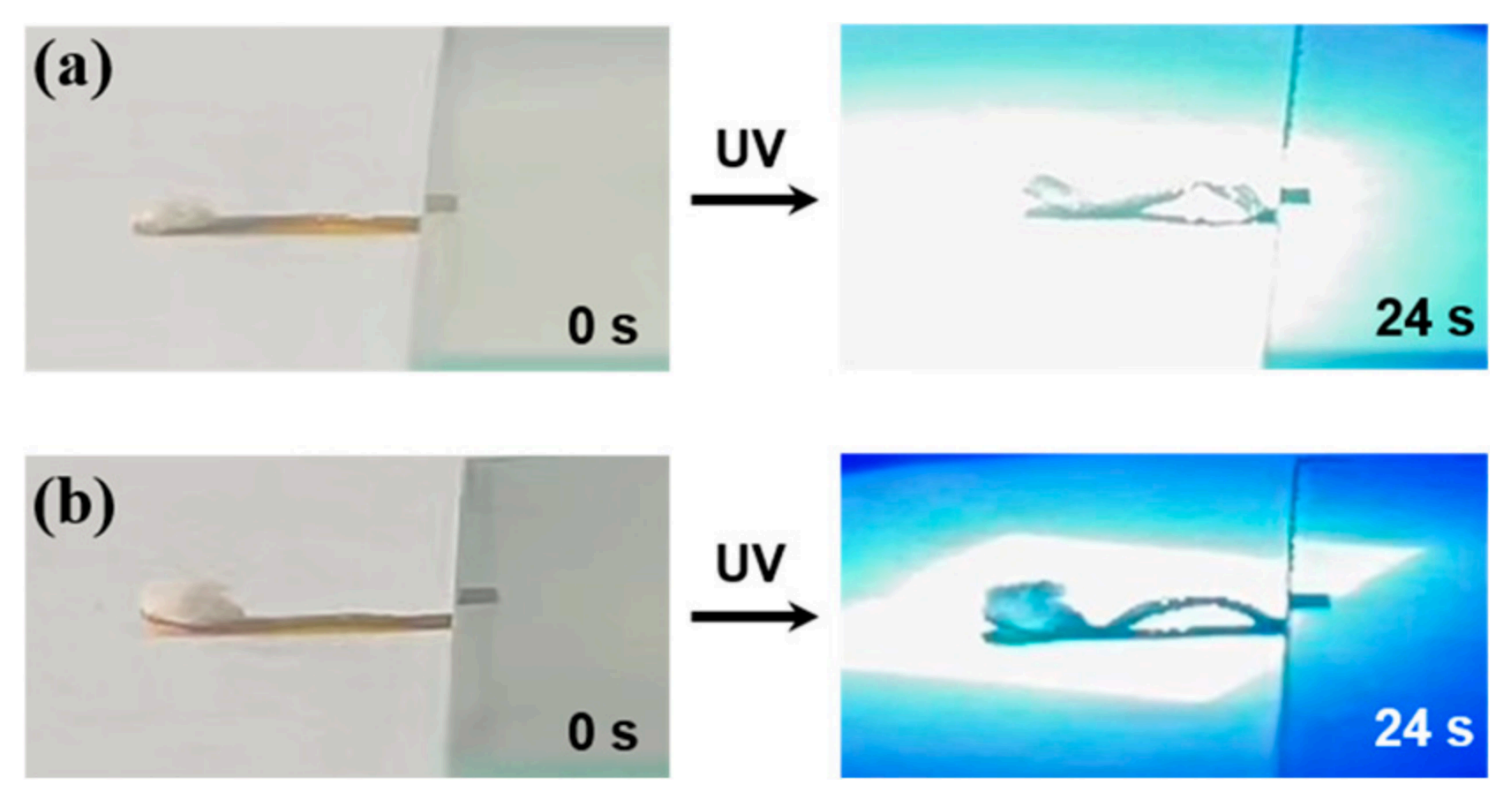
Upon 365 nm UV irradiation (110 mW cm^−2^), the ~0.9 mg LCP films deform into out-of-plane arches. (**a**) Loaded with ~1 mg; (**b**) loaded with ~0.5 mg. Film dimensions: 10 mm × 2 mm × 15 μm (length × width × thickness).

To evaluate the load-bearing capacity of the azobenzene-containing LCP films, load-bearing tests were performed on two independent films (each ~0.9 mg). Cotton balls (~1.0 mg and ~0.5 mg) were attached to the free ends as dead weights using double-sided tape, while the opposite ends were fixed with glass coverslips. Upon 365 nm UV irradiation (110 mW cm^−2^), neither film bent towards the light source. Instead, the loaded end lifted marginally and the midsection of each film buckled into an out-of-plane arch (Figure 7). This behavior is attributed to the loading configuration: the cotton-ball weight at the free end prevents free bending, effectively creating a clamped-clamped boundary condition (both ends fixed). When the irradiated top surface attempts to contract due to the trans-to-cis isomerization of the azobenzene moieties, the constrained ends force the deformation into an out-of-plane arch rather than a cantilever bend. Even at the lower load (~0.5 mg), the film could not overcome the weight to produce bending, indicating that the maximum load-bearing capacity of the LCP film is less than half its own mass (~0.45 mg).

### 2.5. Effect of Light Intensity and Film Thickness on the Photoinduced Deformations of LCP Films

**Figure 8:**
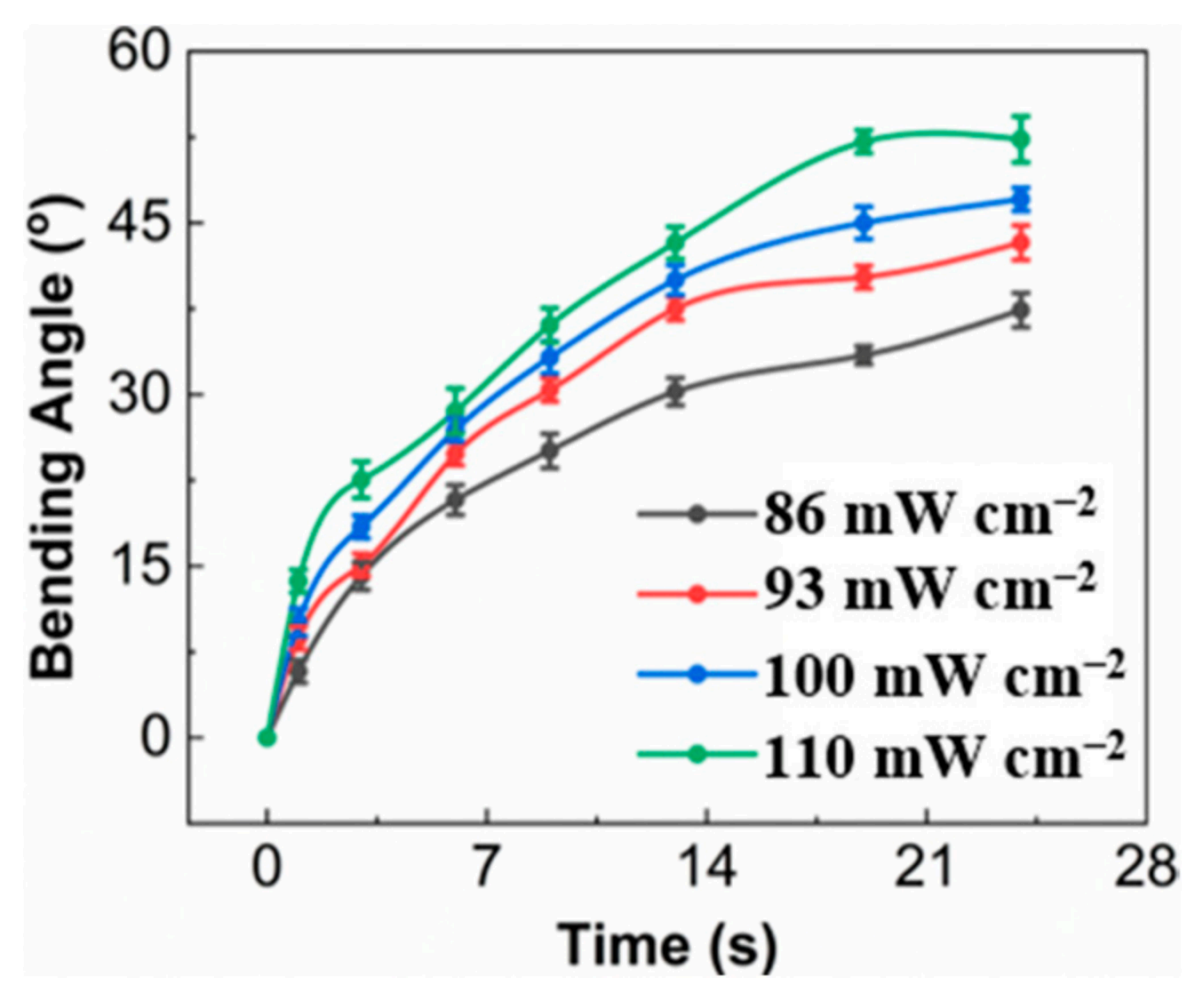
Time-dependent bending angles of the azobenzene-based LCP films at different 365 nm UV intensities.

Figure 8 shows time-dependent bending angles of the azobenzene-containing LCP films at different 365 nm UV intensities. For a given exposure time, a higher irradiation intensity produces a larger bending angle. This trend is attributed to the enhanced trans-to-cis isomerization of the azobenzene moieties on the illuminated surface at higher irradiance, which increases the through-thickness stress gradient and thus yields larger bending angles.

**Figure 9:**
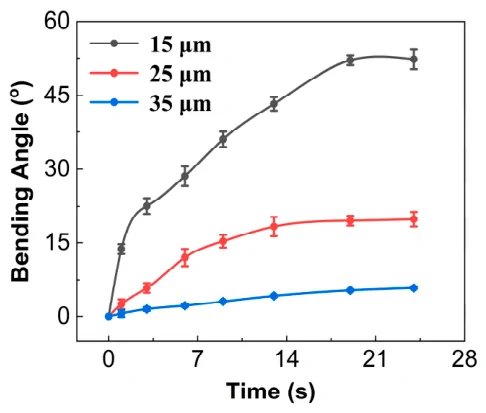
Time-dependent bending angles of the azobenzene-based LCP films with different thicknesses.

Figure 9 shows time-dependent bending angles of the azobenzene-containing LCP films with different film thicknesses. For a given irradiation time and a constant light intensity, the bending angle significantly decreases with increasing film thickness. This result can be ascribed to two combined effects: (i) the flexural stiffness increases with thickness, making the film harder to bend under the same driving stress, and (ii) the penetration depth of 365 nm UV light remains nearly constant, so the relative fraction of the isomerized layer decreases in thicker films, reducing the effective through-thickness stress gradient. Consequently, thicker films exhibit smaller bending angles.

### 2.6. Durability of the Azobenzene-Based LCP

**Figure 10:**
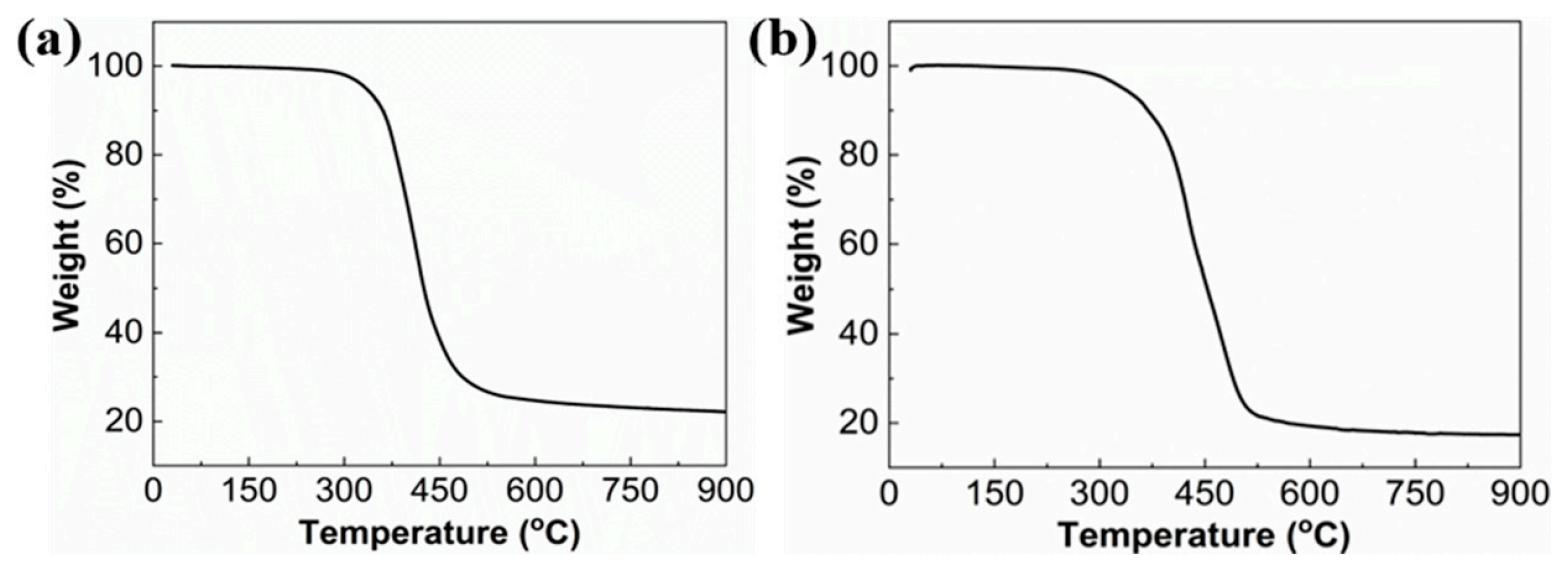
TGA curves of the azobenzene-based LCP under an N_2_ atmosphere (10 °C min^−1^). (**a**) Untreated sample; (**b**) sample subjected to simulated aging—365 nm UV irradiation (110 mW cm^−2^, 15 min during dissolution + 30 min during solvent evaporation) followed by oven drying at 80 °C for 12 h.

The durability under combined photo- and thermal exposure is critical for practical LCP actuators. To evaluate thermal stability, we performed thermogravimetric analysis (TGA) on an untreated sample and a sample subjected to simulated aging: (i) 365 nm UV irradiation (110 mW cm^−2^, 15 min) during dissolution; (ii) continuous UV exposure (110 mW cm^−2^, 30 min) during solvent evaporation; and (iii) oven drying at 80 °C for 12 h. As shown in Figure 10, the decomposition temperature (*T_d_*, 5% weight loss) of the untreated sample was 334.5 °C, while that of the UV/heat-treated sample was 333.1 °C—a difference of only ~1.4 °C, which falls within experimental uncertainty. This indicates that prolonged UV exposure and heating did not cause detectable chemical decomposition of the azobenzene-based LCP. Notably, TGA probes backbone stability rather than photomechanical fatigue; however, the fact that the backbone remained intact after combined UV/heat treatment supports the material’s robustness under operating conditions. In conjunction with the cycling data (Figure 6c), these results collectively demonstrate reasonable durability of the azobenzene-based LCP for photoactuation applications.

## 3. Materials and Methods

### 3.1. Synthesis of Azobenzene-Based Main-Chain LCP

#### 3.1.1. Synthesis of DHAZ

*p*-Aminophenol (10.90 g, 0.10 mol) was dissolved in 13 wt% HCl (90 mL) and cooled in an ice bath. A solution of NaNO_2_ (10.35 g, 0.15 mol) in H_2_O (52 mL) was added dropwise, and the mixture was stirred for 30 min. Subsequently, a solution of phenol (9.40 g, 0.10 mol) and NaOH (10.00 g, 0.25 mol) in H_2_O (120 mL) was added dropwise to the diazonium solution, and the resulting mixture was stirred for 2 h. After completion, the pH was adjusted to 5–6 with 10 wt% HCl, and the reddish-brown precipitate was collected by filtration. The crude product was purified by silica gel column chromatography (ethyl acetate/*n*-hexane = 3:1, *v*/*v*) to afford DHAZ as a brick-red powder. Its ^1^H NMR spectrum is shown in Figure S6.

#### 3.1.2. Synthesis of BECPAZ

DHAZ (4.50 g, 0.02 mol) was dissolved in DMF (40 mL). K_2_CO_3_ (5.82 g, 0.04 mol), ethyl 6-bromohexanoate (14.12 g, 0.06 mol), and KI (1.83 g, 0.01 mol) were added, and the mixture was stirred at 80 °C for 24 h. After cooling to room temperature, the reaction mixture was poured into H_2_O (700 mL) to precipitate the product, which was collected by filtration and dried to give a reddish-brown solid. The crude product was purified by silica gel column chromatography (CH_2_Cl_2_/ethyl acetate = 70:1, *v*/*v*) to afford BECPAZ as an orange-red powder. Its ^1^H NMR spectrum is shown in Figure S7.

#### 3.1.3. Synthesis of BCPAZ

BECPAZ (1.49 g, 3.00 mmol) was dispersed in anhydrous methanol (140 mL). A solution of KOH (2.89 g, 0.05 mol) in H_2_O (35 mL) was added dropwise, and the mixture was heated under reflux for 24 h. After completion, the methanol was removed under reduced pressure by rotary evaporation. The residue was dissolved in H_2_O (200 mL), and insoluble impurities were filtered off. The aqueous phase was acidified to pH 5–6 with 10 wt% HCl, and the resulting yellow precipitate was collected by filtration. The solid was washed several times with H_2_O and dried under vacuum at 50 °C for 24 h to afford BCPAZ as a yellow powder. Its ^1^H NMR spectrum is shown in Figure S8.

#### 3.1.4. Synthesis of the LCP

2-methyl-1,4-bis[4-(oxirane butoxy)benzoyloxy]benzene (0.28 g, 0.50 mmol) and BCPAZ (0.22 g, 0.50 mmol) were dispersed in anhydrous DMF (1 mL) and heated at 140 °C for 5 h. Subsequently, 1,5,7-triazabicyclo[4,4,0]dec-5-ene (7.00 mg, 0.05 mmol) was added, and the reaction was maintained at 140 °C for an additional 19 h. The whole process was carried out under N_2_. After cooling to room temperature, the mixture was added dropwise into ice-cold methanol, precipitating a brownish-yellow solid, which was collected by filtration, washed several times with methanol, and dried under vacuum at 45 °C for 24 h to afford the brownish-yellow LCP.

### 3.2. Preparation of Free-Standing Azobenzene-Based LCP Films

A representative 15 μm-thick free-standing azobenzene-containing LCP film was prepared as follows. The LCP (30.0 mg) was dissolved in DMF (1.5 mL) and drop-cast onto Teflon substrates. After standing at room temperature for 10 h to allow preliminary solvent evaporation, the films were dried in an oven at 80 °C for 10 h. After cooling to room temperature, brownish-yellow films (~15 μm thickness) were obtained by peeling off the substrates.

### 3.3. Characterizations

The structures of the azobenzene-containing intermediates were confirmed by ^1^H NMR spectroscopy (DRX-500, Bruker, Billerica, MA, USA) in DMSO-*d*_6_ or CD_2_Cl_2_ with tetramethylsilane (TMS) as the internal standard. UV-visible absorption spectra of the azobenzene-based LCP upon light irradiation were recorded using a spectrophotometer (Lambda 750, PerkinElmer, Waltham, MA, USA). TGA was performed on a TGA 4000 system (PerkinElmer, Waltham, MA, USA) under a N_2_ atmosphere at a heating rate of 10 °C min^−1^. The thermal transition of the UV-irradiated LCP was measured by DSC (DSC 8000, PerkinElmer, Waltham, MA, USA) under N_2_ at 10 °C min^−1^. Dynamic mechanical properties of the irradiated LCP were measured using a rotational rheometer (MCR 301, Anton Paar, Graz, Austria). LC texture and phase transitions of the LCP, and macroscopic alignment of the LCP film were observed by POM (Axio Scope A1, ZEISS, Oberkochen, Germany). Wide-angle X-ray scattering (WAXS) measurement was performed on a SaxsLab GANESHA system (Cu K*α*, *λ* = 0.154 nm). Surface temperature evolution of the LCP film upon light exposure was monitored using an infrared thermal imager (SC 7000, FLIR, Wilsonville, Oregon, USA). Actuation lights at 365 nm and 530 nm were provided by a controller (HTLD-4, Height-LED, Shenzhen, China), and the corresponding power densities were calibrated by a photometer (CEL-NP2000, Beijing Zhongjiao Jinyuan Technology, Beijing, China). LCP films were heated on a JF-956A heating stage (Dongguan Chang’an Jin Feng Electronic Tools, Dongguan, China).

## 4. Conclusions

In summary, an azobenzene-containing main-chain LCP was synthesized via step-growth polymerization, and free-standing LCP films were prepared by one simple drop-coating method. The LCP exhibited excellent film-forming ability without the need for any pre-alignment or crosslinking steps during film formation. UV-visible absorption spectra demonstrated that alternating 365 nm UV (110 mW cm^−2^) and 530 nm green light (110 mW cm^−2^) induced reversible trans-cis isomerization of the LCP. Rheological measurements at 25 °C revealed a significant switch in *G*′ from 1.7 × 10^7^ to 6.9 × 10^4^ Pa after 365 nm UV irradiation (110 mW cm^−2^) and a partial recovery to 7.2 × 10^5^ Pa after subsequent 530 nm green light exposure (110 mW cm^−2^), directly demonstrating photo-mechanical energy conversion within the LCP. Furthermore, the LCP film bent towards 365 nm UV light (110 mW cm^−2^) with a maximum bending angle of 53.7 ± 2.1° and nearly reverted to the initial flat state (6.5 ± 1.5°) upon exposure to 530 nm green light (110 mW cm^−2^), with reversible bending and flattening over multiple cycles. Systematic studies further revealed that the photoinduced deformations of the LCP films are highly tunable: increasing UV intensity from 86 to 110 mW cm^−2^ enhanced the maximum bending angle from 37.4 ± 1.5° to 53.7 ± 2.1°, while increasing film thickness from 15 to 35 μm suppressed it from 53.7 ± 2.1° to 5.8 ± 0.4°. This alignment-free, solution-processable strategy provides a straightforward route to free-standing azobenzene-based LCP films with predictable, intensity- and thickness-dependent photo-mechanical performance, holding promise for soft robots and light-controlled devices.

## Data Availability

The original data presented in this study are included in the article and Supplementary Material. Raw data are available from the corresponding authors upon reasonable request.

## Supplementary Materials

The following supporting information can be downloaded at: https://www.mdpi.com/article/10.3390/molecules31203692/s1, Scheme S1. Schematic illustration of the preparation process of the LCP films; Figure S1. UV-visible absorption spectrum of the pristine LCP upon 530 nm green light irradiation (110 mW cm^−2^); Figure S2. DSC thermograms of the LCP after 365 nm UV irradiation (110 mW cm^−2^) (a) First measurement, (b) second measurement, (c) third measurement; Figure S3. DSC thermogram of the LCP after sequential 365 nm UV (110 mW cm^−2^) and 530 nm green-light irradiation (110 mW cm^−2^); Figure S4. Upon sequential exposure to 365 nm UV (110 mW cm^−2^) and 530 nm green light (110 mW cm^−2^), the LCP film bends towards the light source and nearly reverts to its initial flat shape. The film dimensions are 10 mm × 2 mm × 15 μm (length × width × thickness); Figure S5. The LCP films show no deformations when heated from room temperature to 35 °C in the dark. (a) Upper surface and (b) bottom surface in contact with the heat source. Film dimensions: 10 mm × 2 mm × 15 μm (length × width × thickness); Figure S6. ^1^H NMR spectrum of DHAZ (500 MHz, DMSO-*d*_6_); Figure S7. ^1^H NMR spectrum of BECPAZ (500 MHz, CD_2_Cl_2_); Figure S8. ^1^H NMR spectrum of BCPAZ (500 MHz, DMSO-*d*_6_); Video S1. The LCP film bends towards the light source with a maximum bending angle of 52.3° upon 365 nm UV irradiation (110 mW cm^−2^); Video S2. The bent LCP film nearly returns to its original flat shape upon 530 nm green light exposure (110 mW cm^−2^).
