# Supplementary material for: Photoinduced Reversible Deformations of Azobenzene-Based Main-Chain Liquid Crystalline Polymer Films
Source: Molecules. 2026 Oct 15;31(20):3692. doi: 10.3390/molecules31203692 (PMC13707893; doi:10.3390/molecules31203692)
Supplement: Supplementary file 1 [file molecules-31-03692-s001.zip › 26X10SI.pdf]

Supporting information for

Photoinduced Reversible Deformations of Azobenzene-Based Main-Chain Liquid Crystalline Polymer Films

Peng Zhang [1,2], Guanfeng Tian [1], Guojie Wang [3,*] and Haifeng Yu [2,*]

[1] College of Chemistry and Chemical Engineering, Dezhou University, Dezhou 253023, China
[2] Key Laboratory of Polymer Chemistry and Physics of Ministry of Education, School of Material Science and Engineering, Peking University, Beijing 100871, China
[3] School of Materials Science and Engineering, University of Science and Technology Beijing, Beijing 100083, China
* Correspondence: guojie.wang@mater.ustb.edu.cn (G.W.); yuhaifeng@pku.edu.cn (H.Y.)

1. UV-visible absorption spectrum of the LCP

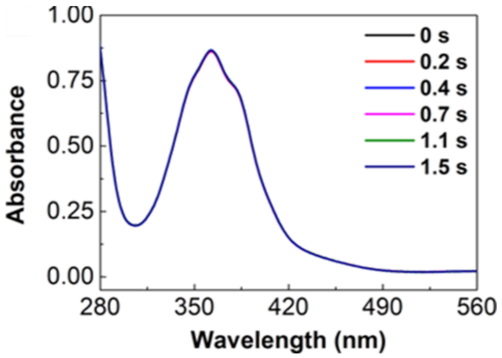

Figure S1. UV-visible absorption spectrum of the pristine LCP upon 530 nm green light irradiation (110 mW cm^{-2}).

---

2. DSC of the LCP

The LCP was dissolved in CH_2Cl_2 (10 wt%). For the *cis*-state sample, the solution was irradiated with 365 nm UV light (110 mW cm^{-2}, 10 min), after which the solvent was evaporated under continuous UV irradiation (110 mW cm^{-2}, 20 min). The resulting sample was dried under vacuum at room temperature for 25 min to ensure complete removal of residual CH_2Cl_2. A ~4 mg sample was then immediately loaded into a sealed Al pan, and DSC measurements were performed in N_2 at a heating rate of 10 °C min^{-1}.

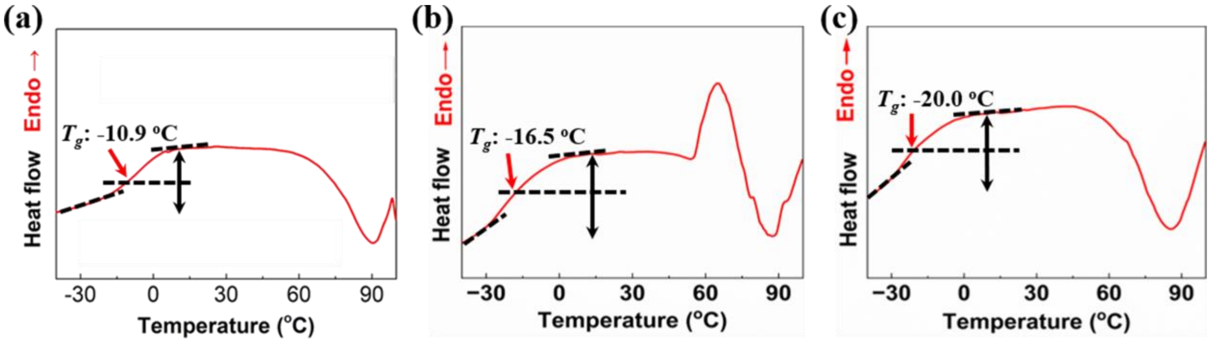

Figure S2. DSC thermograms of the LCP after 365 nm UV irradiation (110 mW cm^{-2}). (a) First measurement, (b) second measurement, (c) third measurement.

The LCP was dissolved in CH_2Cl_2 (10 wt%), irradiated with 365 nm UV light (110 mW cm^{-2}, 10 min), then with 530 nm green light (110 mW cm^{-2}, 10 min), followed by solvent evaporation under continuous green-light irradiation (110 mW cm^{-2}, 20 min). A ~4 mg sample

was immediately loaded into a sealed Al pan and measured by DSC under N_2 at a heating rate of 10 °C min^{-1}.

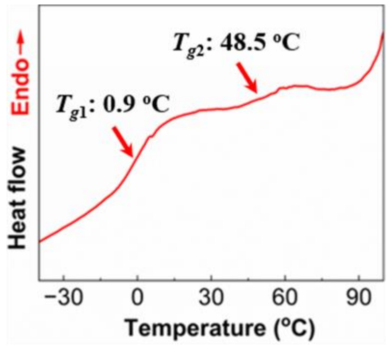

Figure S3. DSC thermogram of the LCP after sequential 365 nm UV (110 mW cm^{-2}) and 530 nm green-light irradiation (110 mW cm^{-2}).

---

3. Preparation of the LCP films

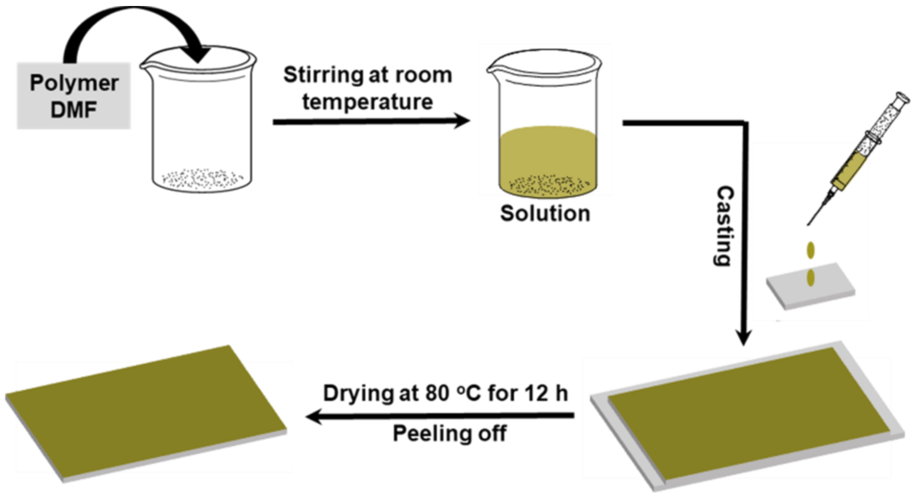

Scheme S1. Schematic illustration of the preparation process of the LCP films.

4. Photoinduced deformations of the LCP film

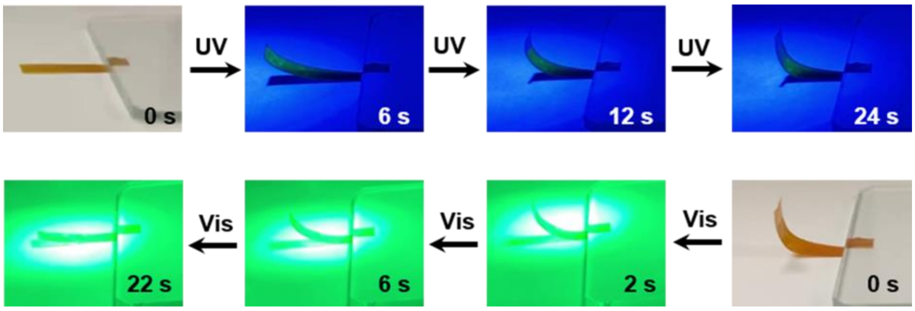

Figure S4. Upon sequential exposure to 365 nm UV (110 mW cm^{-2}) and 530 nm green light (110 mW cm^{-2}), the LCP film bends towards the light source and nearly reverts to its initial flat shape. The film dimensions are 10 mm × 2 mm × 15 μm (length × width × thickness).

---

5. Thermally-induced deformations of the LCP films

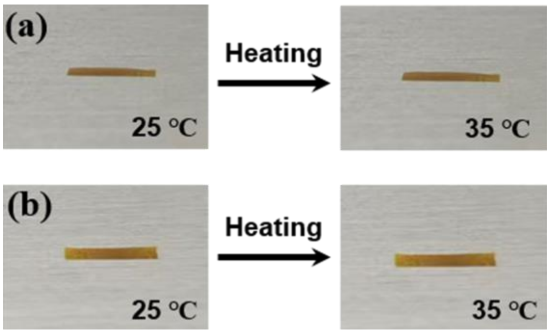

Figure S5. The LCP films show no deformations when heated from room temperature to 35 °C in the dark. (a) Upper surface and (b) bottom surface in contact with the heat source. Film dimensions: 10 mm × 2 mm × 15 μm (length × width × thickness).

6. ^{1}H NMR spectra of the azobenzene-containing intermediates

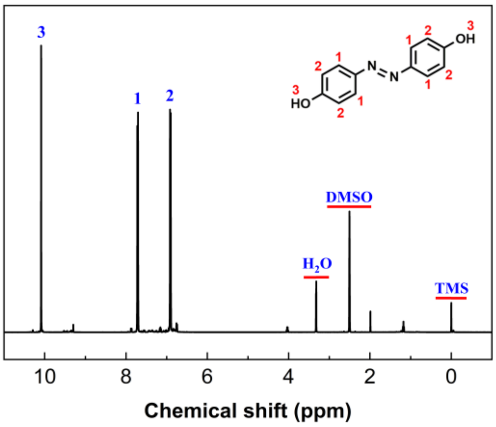

Figure S6. ^{1}H NMR spectrum of DHAZ (500 MHz, DMSO-d_6).

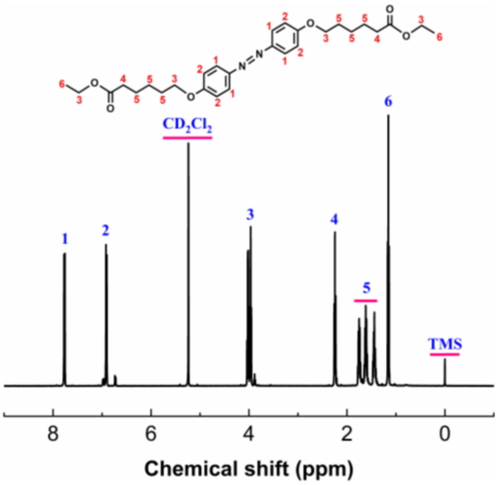

Figure S7. ^{1}H NMR spectrum of BECPAZ (500 MHz, CD_2Cl_2).

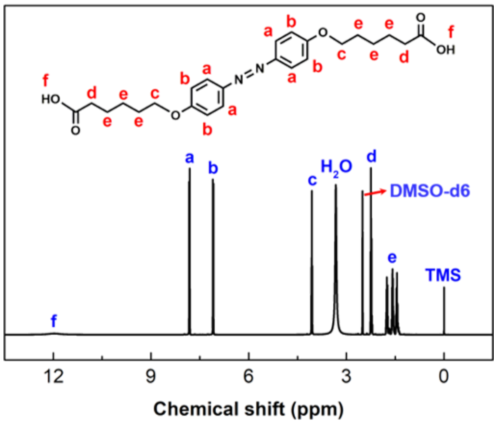

Figure S8. ^{1}H NMR spectrum of BCPAZ (500 MHz, DMSO-d_6).

7. Supplementary videos

Video S1: The LCP film bends towards the light source with a maximum bending angle of 52.3° upon 365 nm UV irradiation (110 mW cm^{-2}).

Video S2: The bent LCP film nearly returns to its original flat shape upon 530 nm green light exposure (110 mW cm^{-2}).
